# Content of intrinsic disorder influences the outcome of cell-free protein synthesis

**DOI:** 10.1038/srep14079

**Published:** 2015-09-11

**Authors:** Alexander A. Tokmakov, Atsushi Kurotani, Mariko Ikeda, Yumiko Terazawa, Mikako Shirouzu, Vasily Stefanov, Tetsuya Sakurai, Shigeyuki Yokoyama

**Affiliations:** 1 Research Center for Environmental Genomics, Kobe University, Nada 657-8501, Japan; 2 RIKEN Systems and Structural Biology Center, Yokohama 230-0045, Japan; 3 RIKEN Center for Sustainable Resource Science, Yokohama 230-0045, Japan; 4 RIKEN Center for Life Science Technologies, Yokohama 230-0045, Japan; 5 Department of Biochemistry, Saint-Petersburg State University, St. Petersburg 199034, Russia; 6 RIKEN Structural Biology Laboratory, Yokohama 230-0045, Japan

## Abstract

Cell-free protein synthesis is used to produce proteins with various structural traits. Recent bioinformatics analyses indicate that more than half of eukaryotic proteins possess long intrinsically disordered regions. However, no systematic study concerning the connection between intrinsic disorder and expression success of cell-free protein synthesis has been presented until now. To address this issue, we examined correlations of the experimentally observed cell-free protein expression yields with the contents of intrinsic disorder bioinformatically predicted in the expressed sequences. This analysis revealed strong relationships between intrinsic disorder and protein amenability to heterologous cell-free expression. On the one hand, elevated disorder content was associated with the increased ratio of soluble expression. On the other hand, overall propensity for detectable protein expression decreased with disorder content. We further demonstrated that these tendencies are rooted in some distinct features of intrinsically disordered regions, such as low hydrophobicity, elevated surface accessibility and high abundance of sequence motifs for proteolytic degradation, including sites of ubiquitination and PEST sequences. Our findings suggest that identification of intrinsically disordered regions in the expressed amino acid sequences can be of practical use for predicting expression success and optimizing cell-free protein synthesis.

Cell-free heterologous protein synthesis is commonly used for production of recombinant proteins. Most often, eukaryotic proteins and their domains are expressed in cell-free bacterial extracts. However, only a fraction of all proteins can be successively expressed in this system as properly folded and functionally active protein products. The rate of soluble expression for natural or partially truncated human proteins in both cell-free bacterial extracts and in *E.coli* bacterial cells was reported to be about 25%^1^,^2^. A higher success rate can be achieved when individual functional domains of heterologous proteins are expressed in the bacterial cell-free system. The main factors reducing expression success in this heterologous system are intrinsic differences between cytoplasmic environments of bacterial and eukaryotic cells and inability of bacterial extracts to support post-translational modifications (PTMs) in eukaryotic proteins. To overcome these difficulties, protein expression systems based on the use of extracts prepared from eukaryotic hosts, such as budding yeast^3^, unicellular flagellate *Leishmania*^4^, insect cells^5^, wheat germ^6^, frog eggs^7^, mammalian cells^8^,^9^,^10^, etc. have been developed.

Various physicochemical and structural features of expressed polypeptides were implicated as determining factors of successful cell-free bacterial expression. They include sequence length, hydrophobicity, solvent accessibility, pI, content of aromatic, nonpolar and charged residues, content of disordered sequences, number of disulfide bonds and structural domains, presence of signaling sequences, PEST regions and transmembrane domains^2^. In addition, it was demonstrated that multiple PTMs affect protein synthesis in this expression system^11^.

Per contra, the factors determining expression success in the eukaryotic systems of protein synthesis have not been investigated in depth so far. To fill this gap, in the present study we examined the correlations of protein expression yields experimentally observed in an insect cell-free expression system with the contents of intrinsic disorder bioinformatically predicted in the expressed sequences. We also compared these correlations with the tendencies witnessed previously in a bacterial system of cell-free protein synthesis. The proteins with long disordered regions, often referred to as intrinsically disordered regions (IDRs), are widespread in eukaryotes. It was estimated that more than one third of eukaryotic proteins contain long IDRs of more than 30 residues in length^12^, and about 25% of the proteins are mostly disordered^13^,^14^. These proteins are highly abundant in the regulatory processes, such as transcription and intracellular signaling^15^,^16^. Amino acid compositional bias represents a common feature of disordered sequence with abundance of charged and hydrophilic amino acids, such as Arg, Gln, Glu, Lys, Ser and underrepresentation of hydrophobic residues, such as Cys, Leu, Ile, Phe, Trp, Tyr, Val^17^. Moreover, IDRs are enriched in disorder-promoting amino acids, resulting in the absence of stable secondary structure and high flexibility, however they often undergo disorder-to-order transition when involved in protein-protein interactions^18^,^19^. Importantly for the outcome of cell-free protein synthesis, IDRs have low hydrophobicity, high net charge and high solvent accessibility^18^,^20^. All these features were shown to be associated with increased protein solubility. The negative correlation of soluble protein expression with hydrophobicity, as well as the positive correlation with net charge and solvent accessibility have been observed previously in a bacterial cell-free expression system^2^. Furthermore, high content of intrinsic disorder was associated with increased soluble expression in this system.

Importantly, disordered regions of proteins are often targeted for multiple PTMs and proteolytic intervention because of their high accessibility. A well-known example of PTM that occurs predominantly in the disordered regions of eukaryotic and plant proteins includes phosphorylation^21^,^22^,^23^,^24^. In addition, a number of statistically significant correlations between intrinsic disorder and glycosylation, acetylation, methylation, ubiquitination and some other PTMs have been revealed^17^,^24^,^25^,^26^,^27^,^28^. In most cases, the investigated PTMs displayed a preference for occurrence in the disordered regions of proteins. High susceptibility of IDRs to post-translational modifications may be directly related to the expression success of heterologous cell-free protein synthesis because multiple PTMs were shown to affect this process^11^. In addition, susceptibility of IDRs to proteolytic intervention may also have an adverse effect on protein amenability to cell-free protein expression.

In this work, we report that elevated disorder content is associated with the increased ratio of soluble expression and decreased propensity for total detectable protein expression in a eukaryotic system of heterologous cell-free protein synthesis. We further demonstrate that these tendencies can be attributed to the specific features of intrinsically disordered regions, such as low hydrophobicity, high accessibility and abundance of sequence motifs for proteolytic degradation.

## Results and Discussion

### Gross estimation of protein expression

**Figure 1:**
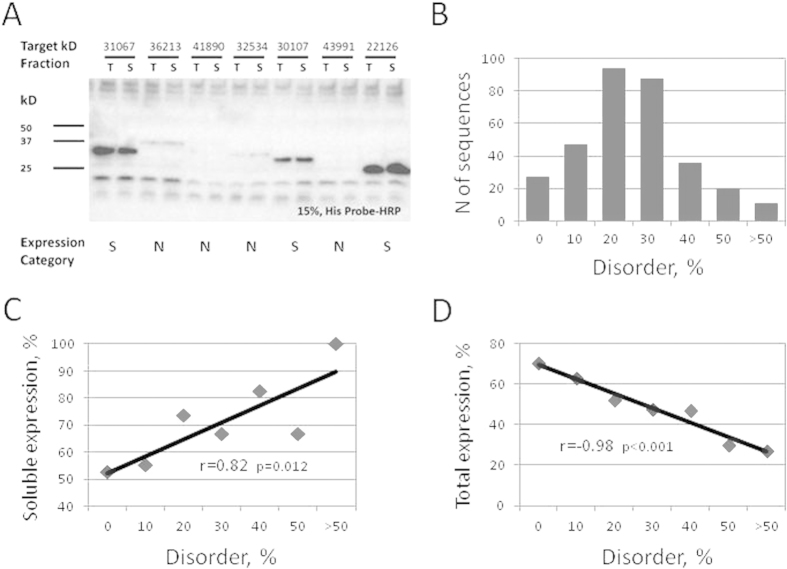
Correlations of cell-free protein expression with contents of intrinsic disorder. Three hundred twenty three (323) non-redundant human amino acid sequences were expressed in an insect cell-free system of protein synthesis under the uniform set of conditions and detected by Western blot analysis. A representative blot is shown in panel (**A**). The sequences were classified into the three expression categories – soluble (S), insoluble (I) and non-expressed (N). Distribution of the dataset proteins according to disorder contents is presented in panel (**B**). Positive correlation of soluble protein expression with disorder degree (**C**) and negative correlation of total detectable protein expression with disorder degree (**D**) were observed in the expression dataset. Pearson’s pairwise correlation coefficients and their statistical significance are indicated in panels (**C**,**D**).

A complete dataset of human proteins and their domains analyzed in this study comprised 323 non-redundant (at 90% level of identity and coverage) amino acid sequences. All proteins in the dataset were synthesized cell-free under the same uniform set of conditions and detected with anti-His antibody, as described in “Methods”. A representative Western blot is shown in Fig. 1A. After evaluation of protein expression levels, each sequence of the dataset was classified into one of the three expression categories – soluble, insoluble or non-expressed, in accordance with the preferential pattern of its expression. The category of non-expressed sequences included the proteins expressed below the detection threshold as well as the proteins expressed at a lower molecular weight than expected. Global estimation of cell-free protein synthesis showed that soluble-expressed proteins represented 34.4%, insoluble-expressed proteins – 16.7%, and non-expressed proteins – 48.9%. Notably, the rate of soluble expression was higher and the rate of insoluble expression was lower than those reported for a related set of human proteins expressed in a bacterial cell-free expression system (25.7% and 46.7%, respectively^2^). In addition, the rate of non-expressed proteins (48.9%) was higher in the eukaryotic expression system than that observed in the bacterial system (27.6%). These results indicate that, in the investigated systems of heterologous cell-free protein expression, protein synthesis earns better solubility but lower production yield in the eukaryotic system, as compared to those in the bacterial system.

### Correlations of protein expression with intrinsic disorder

In the next step of the analysis, disorder content was calculated for every amino acid sequence in the expression dataset using the prediction algorithm RONN. The prediction accuracy of this tool was reported to be about 85%^29^. Although more than 50 tools for protein disorder prediction have been developed^14^,^30^, RONN was chosen as the main predictor in this study because its scores were closer to the experimental scores of disorder for PDB-deposited protein structures. Following disorder assignment, the correlations between protein expression scores and contents of intrinsic disorder were examined. The rates of soluble and total (soluble and insoluble) detectable protein expression were determined at different values of disorder content, covering the entire parameter range observed in the analyzed dataset (from 0% to ≥50%), with the average content of disorder in the analyzed dataset being ~20.5% (Fig. 1B). A strong statistically significant (p = 0.012) positive correlation between the disorder degree and soluble protein expression was observed in the dataset (Fig. 1C). In addition, a negative correlation between the total detectable protein expression and disorder contents was also evident (Fig. 1D). It had a high statistical significance (p < 0.001) too. These results are consistent with the data previously obtained in a bacterial cell-free expression system. The positive correlations of disorder degree with the rates of soluble and undetectable protein expression were observed in that system^2^. Altogether, the results obtained indicate that protein disorder is associated with increased soluble expression and decreased overall expression propensity in the investigated systems of heterologous cell-free protein synthesis.

### Comparison of the expression-disorder correlations in single-domain and multi-domain proteins

**Figure 2:**
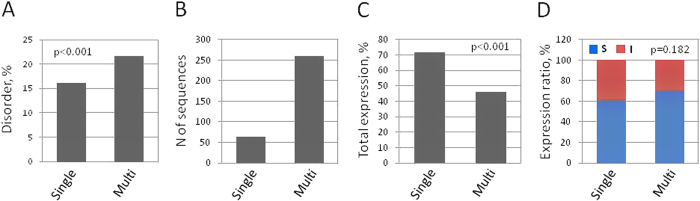
Correlations of protein expression with intrinsic disorder in single-domain and multi-domain proteins. (**A**) Average content of intrinsic disorder in single-domain and multi-domain proteins of the expression dataset, (**B**) total numbers of single-domain and multi-domain proteins in the dataset. Rates of total detectable protein expression and soluble-to-insoluble expression in single-domain and multi-domain proteins of the dataset are presented in panels (**C**,**D**). p-values for the observed differences between subsets of single-domain and multi-domain proteins are indicated in panels (**A**,**C**,**D**).

To confirm these findings, correlations between protein expression and intrinsic disorder were investigated in the single-domain and multi-domain proteins of the experimental dataset. Intrinsically disordered regions are known to serve as the linkers between functional domains in multi-domain proteins, allowing their global flexibility and multiple interactions with various partners^31^. Especially, non-helical linkers were found to be largely unstructured and rich in proline residues^32^. Thus, generally, it can be expected that the content of disordered sequence is elevated in multi-domain proteins in comparison with that in single-domain proteins. Indeed, the average content of disordered sequence was higher in the multi-domain proteins of the expression dataset (Fig. 2A). Moreover, a strong positive correlation (p < 0.001) was observed between disorder content and predicted number of functional domains in the proteins of the analyzed dataset (Fig. S1A). We also confirmed the correlation between intrinsic disorder and domain number by the comprehensive genome-wide analysis of a full human proteome dataset, which contained 22,995 human protein sequences extracted from a NCBI database of human genome (the annotation release 106) downloaded from ftp://ftp.ncbi.nlm.nih.gov/genomes/H_sapiens/protein/. The end result of this analysis is presented in Fig. S1B. It seems to contradict a previous research, reporting that the correlation between intrinsic disorder and the number of ordered domains was poor in non-hub proteins and negative in hubs, defined as the proteins with five or more interactions^33^. It should be noted, however, that the domain predictive algorithms used in the two studies were different. Domain assignment in the previous work was based on a Pfam homology-based algorithm, whereas the DomCut algorithm employed in this study utilizes sequence–specific features for domain prediction. As a result, domain definition is different in the two algorithms, and the outcomes of their predictions are not the same. Indeed, the distributions of multi-domain proteins determined in the expression dataset using these two methods differ significantly (Fig. S2). In this work, the DomCut predictive algorithm was employed because it designates long unstructured pieces of amino acid sequence as the inter-domain linkers, allowing correlation analysis between the domain number and intrinsic disorder.

Importantly for statistical cue, the expression dataset contained sufficient numbers of single- and multi-domain proteins (64 and 259 sequences, correspondingly, Fig. 2B). Analysis of protein expression data showed that the percentage of detectably expressed sequences was significantly (p < 0.001) lower in the subset of multi-domain proteins (Fig. 2C). However, the propensity for soluble expression was higher in this subset, albeit with a low statistical significance (p = 0.182; Fig. 2D). These data are in close agreement with the results presented in Fig. 1, and they support the notion that high content of intrinsic disorder is associated with increased soluble expression and decreased overall expression propensity. Previously, worsening of protein expression with the number of functional domains was observed in a prokaryotic system of cell-free protein synthesis^11^.

### A rationale for the positive correlation between disorder content and soluble protein expression

**Figure 3:**
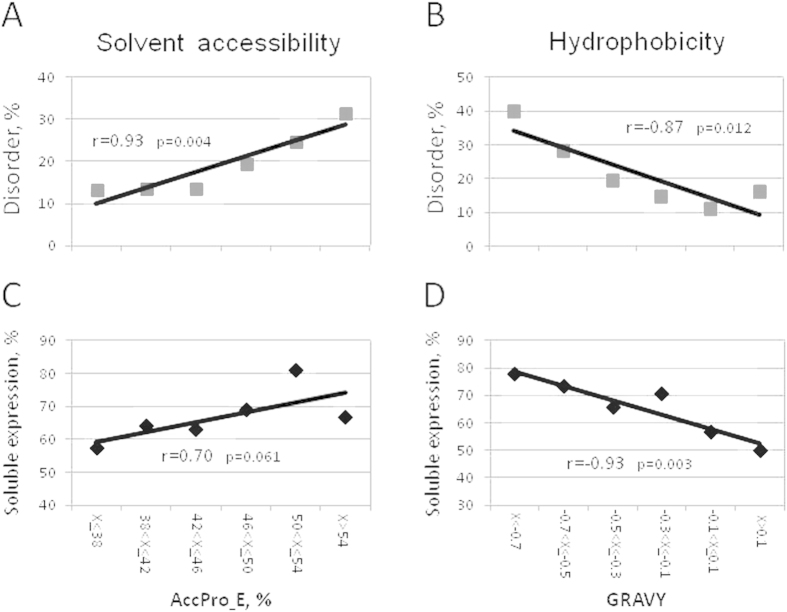
Elevated propensity for soluble expression is associated with high solvent accessibility and low hydrophobicity of IDRs. (**A**) Positive correlation between solvent accessibility and disorder degree, (**B**) negative correlation between hydrophobicity and disorder degree, (**C**) positive correlation between soluble protein expression and solvent accessibility and (**D**) negative correlation between soluble protein expression and disorder degree were observed in the expression dataset. Pearson’s pairwise correlation coefficients and their statistical significance are indicated in the panels.

The data presented in Figs 1C and 2D demonstrate that high content of intrinsic disorder is associated with soluble protein expression. They also suggest that the abundance of disordered sequence should generally promote higher protein solubility. The major factors that can account for the better solubility of proteins with high content of intrinsic disorder may be their elevated surface accessibility and decreased hydrophobicity due to abundance of charged and polar residues^17^,^18^,^20^. These features of disordered regions should affect the integral characteristics (i.e. accessibility and hydrophobicity) of entire protein sequences. A positive correlation between disorder and surface accessibility, as well as a negative correlation between disorder and hydrophobicity, have been observed in the analyzed dataset of expressed sequences (Fig. 3A,B). Previously, both high surface accessibility and low hydrophobicity were found to be associated with increased soluble expression in a bacterial cell-free system of protein synthesis^2^. In the present study, using eukaryotic system of protein synthesis, we also observed similar correlations (Fig. 3C,D). Taken together, the results presented in Figs 1C,2D and 3 suggest that the proteins with high content of intrinsic disorder have an elevated propensity for soluble expression due to their high solvent accessibility and low hydrophobicity.

### A rationale behind the negative correlation between disorder content and overall expression propensity: involvement of ubiquitination

**Figure 4:**
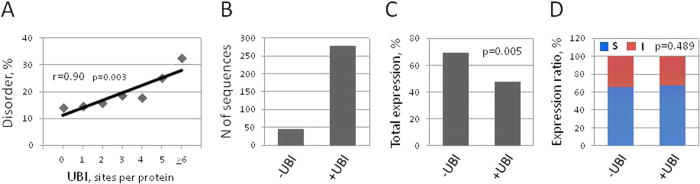
Presence of ubiquitination sites in amino acid sequences decreases their expression propensity. (**A**) Positive correlation between disorder degree and number of ubiquitination sites. Pearson’s pairwise correlation coefficient and its statistical significance are indicated. (**B**) Total numbers of protein sequences with (+UBI) or without (−UBI) predicted sites of ubiquitination in the expression dataset. Panels (**C**,**D**) show the rates of total detectable protein expression and soluble-to-insoluble expression in +UBI and −UBI proteins of the dataset. p-values for the observed differences between subsets of +UBI and −UBI proteins are indicated in the panels.

Next, we have made an effort to find an explanation for the observed negative correlation between disorder contents and total (soluble + insoluble) protein expression (Figs 1D and 2C). This tendency could also be detected in a bacterial system of cell-free protein synthesis^11^. Notably, the intrinsic features of disordered regions, such as high surface accessibility and low hydrophobicity, were found to be associated with low propensity for total detectable protein expression both in bacterial^2^ and eukaryotic (Fig. S3) cell-free expression systems. Considering that the proteins with high content of intrinsic disorder have increased surface accessibility (Fig. 3B) and represent favorable targets for PTMs and proteolytic intervention, we hypothesized that the decreased propensity of disordered sequences for detectable cell-free expression may be related to post-translational protein processing rather than to protein synthesis itself. In this connection, it was demonstrated previously that the proteins with high content of disorder are subject to fast proteolytic degradation by multiple ubiquitin-dependent and -independent protein degradation pathways^34^,^35^. Importantly, ubiquitination was reported to display a clear preference for disordered regions^36^,^37^. We confirmed this finding in the present study. A strong statistically significant (p = 0.003) positive correlation between disorder content and the number of predicted ubiquitination sites was observed in the expression dataset (Fig. 4A). This modification was highly abundant; about 85% of the proteins in the expression dataset contained the predicted site(s) of ubiquitination (Fig. 4B), suggesting high relevance of this PTM in the analyzed expression dataset. Consistently, it was calculated before that more than 70% of proteins in the human proteome have at least one ubiquitination site^36^. We found that the rate of total detectable protein expression was significantly (p = 0.005) lower in the subset of proteins containing predicted sites of ubiquitination, as compared to the subset of ubiquitination-negative proteins (Fig. 4C). Importantly, this tendency could not be observed in a bacterial system of cell-free protein synthesis^11^. The difference between the prokaryotic and eukaryotic expression systems can be attributed to the lack of ubiquitination in bacteria, whereas the insect cell-free protein synthesis system employed in this study was demonstrated to have the potential to perform this PTM^38^. Considering strong positive correlation between disorder content and ubiquitination (Fig. 4A), our data argue that ubiquitin-mediated protein degradation may be a *bona fide* factor behind the negative correlation between intrinsic disorder and overall expression propensity observed in the eukaryotic expression system in this study. Notably, only a little increase of a low statistical significance (p = 0.489) was observed in the ratio of soluble to insoluble expression in the subset of ubiquitination-positive proteins, as compared to the ubiquitination-negative subset (Fig. 4D). Although this result generally agrees with that obtained previously in the bacterial cell-free expression system, a much stronger trend was evidenced in the bacterial system^2^. Altogether, it can be concluded that ubiquitination affects overall expression propensity rather than soluble protein expression, and that the presence of ubiquitination sites in amino acid sequences decreases their overall propensity for detectable expression in the employed eukaryotic system of cell-free protein synthesis.

### Involvement of PEST-mediated protein degradation

**Figure 5:**
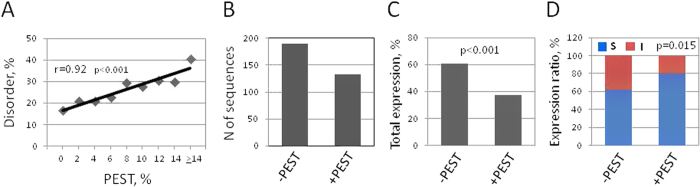
Presence of PEST motifs in amino acid sequences affects their cell-free expression propensity. (**A**) Positive correlation between disorder degree and contents of PEST sequences. Pearson’s pairwise correlation coefficient and its statistical significance are indicated. (**B**) Total numbers of protein sequences with (+PEST) or without (−PEST) predicted PEST motifs in the expression dataset. Panels (**C**,**D**) show the rates of total detectable protein expression and soluble-to-insoluble expression in +PEST and −PEST proteins of the dataset. p-values for the observed differences between subsets of −+PEST and −PEST proteins are indicated in the panels.

Although ubiquitination mediates one of the major proteolytic pathways, some other mechanisms of proteolysis are also involved in protein degradation in eukaryotic cells. It was reported that PEST sequences represent a universal target for proteolytic degradation^39^,^40^. Notably, amino acid compositional bias in IDRs^17^,^41^ suggests that they may be enriched in PEST motifs, however the abundance of PEST sequences in the disordered protein regions has never been addressed in previous studies. Therefore, we investigated whether any correlation exists between PEST and disorder contents. A strong positive correlation was observed between the predicted PEST and disorder contents (p < 0.001) in the analyzed expression dataset (Fig. 5A). This correlation was further confirmed by the comprehensive genome-wide analysis of human proteome (Fig. S4). Similarly to ubiquitination, the presence of PEST sequences in the expression dataset was quite common; about 41% of all the proteins in the dataset were predicted to contain PEST-rich motifs (Fig. 5B). Remarkably, the rate of detectable protein expression was significantly (p < 0.001) lower in the subset of proteins containing PEST sequences, as compared to the subset of PEST-negative proteins (Fig. 5C). In addition, the rate of soluble expression was elevated in the subset of PEST-positive proteins (Fig. 5D), with a high statistical significance (p = 0.015). These data agree well with the results obtained previously in a bacterial system of cell-free protein synthesis, however the revealed trends were more pronounced in the bacterial system^2^. The reason for this may be a higher efficiency of general proteolytic decay (including PEST-targeted degradation) in bacteria, as compared to eukaryotic cells. Eukaryotes developed alternative mechanisms of protein degradation, such as ubiquitin- and proteasome-mediated decay, diminishing the importance of general proteolysis for protein clearance. It was proposed that low-complexity regions of overexpressed mammalian proteins, such as PEST motifs, may be the targets for proteolytic degradation in bacteria because they are less common in bacterial proteins^42^. Thus, we conclude that the presence of PEST motifs decreases overall propensity of amino acid sequences for detectable cell-free expression due to their augmented proteolytic degradation.

In sum, our work demonstrates that the content of intrinsic disorder affects protein amenability to heterologous cell-free expression. Specifically, propensity for soluble expression increases with disorder content. This tendency is rooted in the distinct features of intrinsically disordered regions, such as low hydrophobicity and elevated surface accessibility, the properties commonly associated with elevated protein solubility. In addition, overall propensity for detectable protein expression decreases with disorder content. This trend can be attributed to the fact that disordered regions are enriched in sequence motifs targeting polypeptides for proteolytic degradation, such as the sites of ubiquitination and PEST-containing motifs. Our findings suggest that bioinformatics prediction of disordered regions in the expressed amino acid sequences can be used practically for protein engineering aimed at increasing yield and solubility of cell-free protein synthesis.

## Methods

In detail, the approach aimed at the identification of statistically significant correlations between calculated and predicted properties of amino acid sequences and their amenability to heterologous cell-free expression has been described previously^43^,^44^. It includes: (1) batch-mode screening-scale protein synthesis, (2) categorical assignment of expression scores, (3) calculation and prediction of multiple properties of expressed sequences, (4) correlation of the individual properties with the expression scores and (5) evaluation of statistical significance of the observed correlations. Using this approach, a number of important statistically significant correlations between calculated and predicted properties of amino acid sequences and their propensity for cell-free expression have been revealed^2^,^11^. The individual steps of the developed analysis were implemented in the present study, as detailed below.

### Cell-free protein expression

Cell-free protein synthesis directly from PCR-generated linear DNA fragments was used. Coding sequences for the selected protein targets were amplified by 2-step PCR according to the previously described procedure^45^ from the source human cDNA clones obtained from Invitrogen, Carlsbad, CA, USA; OriGene Technologies, Rockville, MD, USA; Kazusa DNA Research Institute, Kisarazu, Chiba, Japan; Institute of Medical Science of Tokyo University, Tokyo, Japan; GeneCopoeia Inc., Rockville, MD, USA; and Toyobo Engineering, Osaka, Japan. Primer oligonucleotides were purchased from Invitrogen and Sigma-Genosys (Woodlands, TX, USA). The resulting linear DNA templates for cell-free protein synthesis universally comprised the sequences encoding the N-terminal T7 RNA polymerase promoter, polyhedrin 5′ UTR and poly-His tag, as well as the C-terminal 3′ UTR, poly A and T7 RNA polymerase terminator sequences. The linear DNA templates were transcribed *in vitro* with T7 RNA polymerase using the ScriptMAX kit (Toyobo, Osaka, Japan) according to the manufacture’s instruction. The transcripts were purified using MicroSpin G-25 Columns (GE) and their absorbance was measured at 260 and 280 nm to determine purity and concentration. Cell-free protein synthesis was carried out using the Transdirect insect cell-free expression system according to the manufacturer’s manual (Shimadzu, Kyoto, Japan). Protein synthetic reactions were performed at 25 °C for 5 h in a final volume of 30 μl. The screening for expressed recombinant proteins was carried out in a 96-well format to allow simultaneous processing of multiple samples. Aliquots of protein synthetic reactions were transferred to Eppendorf tubes, then soluble and insoluble products of protein synthetic reactions were separated by centrifugation at 10,000 g for 10 min. Although this procedure cannot discriminate between protein aggregates and genuinely soluble proteins, it provides the upper estimation of soluble expression. Five-μl aliquots of total and supernatant fractions were subjected to SDS-PAGE on 15% gels and detected by Western blot analysis using a HisProbe-HRP kit for His-tagged protein detection (Pierce Biotechnology, Rockford, IL). The protein expression level and solubility were estimated by quantifying the intensities of specific bands in the total and supernatant fractions.

### Calculation and prediction of multiple properties of expressed sequences

Multiple features of the amino acid sequences in the expression dataset were calculated and predicted using existing bioinformatics tools. Content of disordered structure was predicted with the RONN software^29^ available online (http://www.strubi.ox.ac.uk/RONN). Interdomain linkers were predicted with the DomCut tool^46^ provided online at http://www.bork.embl.de/~suyama/domcut/ and functional domains were predicted with HMMPfam tool of InterProScan 5^47^ downloaded from http://www.ebi.ac.uk/interpro/download.html. The sites of ubiquitination were predicted using the site-specific predictor UbPred^48^ freely downloadable for academic research from http://ubpred.org/. Solvent accessibility was assessed with the ACCpro 4.0 software downloaded from the SCRATCH Protein Predictor server^49^ (http://scratch.proteomics.ics.uci.edu/explanation.html). The grand average of hydropathicity (GRAVY) index was calculated using free software available at the Expasy server (http://web.expasy.org/protparam/) and PEST sequences (sequences rich in P, E, S, and T) were predicted with a tool provided online (http://emboss.bioinformatics.nl/cgi-bin/emboss/epestfind).

### Categorical data evaluation

A complete expression dataset analyzed in this study comprised 323 non-redundant human amino acid sequences. The redundancy level was evaluated with the OrthoMCL tool^50^ (orthomcl.org/cgi-bin/OrthoMclWeb.cgi?rm = orthomcl#Software), and it was set at 90% sequence identity at more than 90% of sequence coverage. Proteins of different functional classes were represented in the dataset. Based on the results of cell-free expression, all proteins in the dataset were classified into the three expression categories – soluble (S), insoluble (I) and non-expressed (N). Each protein sequence could only be placed into one expression category. In the case when expressed polypeptide was found in both soluble and insoluble fractions of cell-free extract, it was categorized according to the preferential pattern of its expression, as judged by lane-to-lane comparison of total and supernantant fractions of the extract on SDS PAGE (for details, see “Cell-free protein expression” section). The polypeptides expressed at a lower molecular weight than expected were classified into the category of non-expressed proteins, because they could not attain proper structure and function when synthesized in this cell-free system. They constituted about 9.5% of all non-expressed proteins.

### Correlation and statistical analyses

Correlations between the expression scores and continuous variables (i.e. calculated or predicted parameters of amino acid sequences), such as hydrophobicity, solvent accessibility and PEST content, were analyzed by determining percentage of sequences in the corresponding expression categories (S, I and N) at different values of analyzed variables. These values covered the entire parameter range observed in the expression dataset. Pearson’s pairwise correlation coefficients were determined and their statistical significance was evaluated by calculating one-tailed probability values, given the value of correlation coefficient, sample size and confidence level. It was set to 0.95 in this study. The variables that can adopt only a finite number of possible values, such as the number of ubiquitination sites and number of functional domains, were processed as the Yes/No features, which can be either present in or absent from a sequence. The two-way contingency table test was applied to evaluate statistical significance of differences between the Yes and No subsets of expressed sequences^51^. The Fisher’s exact p-values were determined using a statistics tool provided online. Calculations of both correlation coefficients and *p*-values were performed using the online statistics calculators available at http://www.danielsoper.com/statcalc3/.

## Additional Information

**How to cite this article**: Tokmakov, A. A. *et al.* Content of intrinsic disorder influences the outcome of cell-free protein synthesis. *Sci. Rep.* **5**, 14079; doi: 10.1038/srep14079 (2015).

## Supplementary Material

Supplementary Information
