# Supplementary material for: Content of intrinsic disorder influences the outcome of cell-free protein synthesis
Source: Sci Rep. 2015 Sep 11;5:14079. doi: 10.1038/srep14079 (PMC4566126; doi:10.1038/srep14079)
Supplement: Supplementary Information [file srep14079-s1.pdf]

Supplementary information

for MS SREP-15-03787

Content of intrinsic disorder influences the outcome of cell-free protein synthesis

Alexander A. Tokmakov[1,2*], *Atsushi Kurotani*[3], *Mariko Ikeda*[2,4], *Yumiko Terazawa*[2], *Mikako Shirouzu*[2,4], *Vasily Stefanov*[5], *Tetsuya Sakurai*[3] & *Shigeyuki Yokoyama*[2,6]

[1]*Research Center for Environmental Genomics, Kobe University, Nada 657-8501, Japan;* [2]*RIKEN Systems and Structural Biology Center, Yokohama 230-0045, Japan;* [3]*RIKEN Center for Sustainable Resource Science, Yokohama 230-0045, Japan;* [4]*RIKEN Center for Life Science Technologies, Yokohama 230-0045, Japan;* [5]*Department of Biochemistry, Saint-Petersburg State University, St. Petersburg 199034, Russia;* [6]*RIKEN Structural Biology Laboratory, Yokohama 230-0045, Japan*

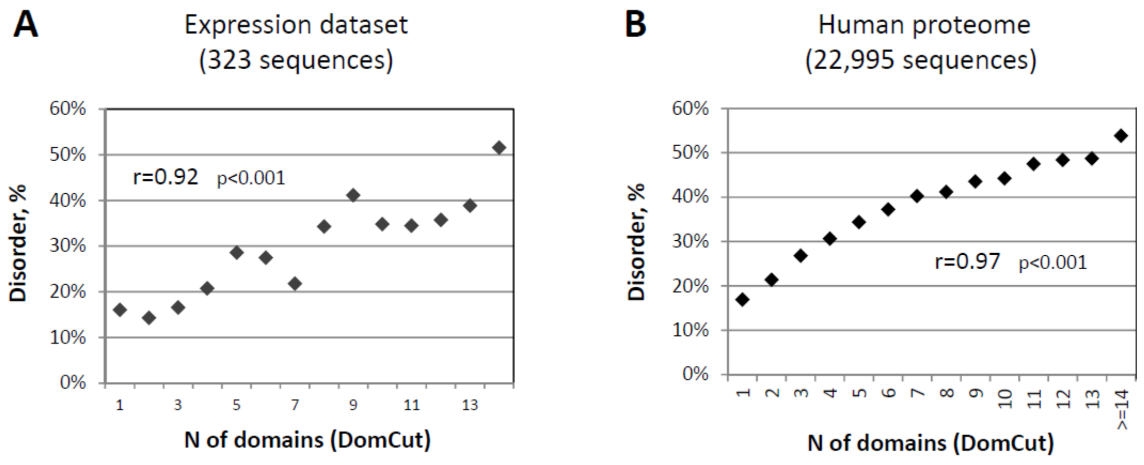

Figure S1

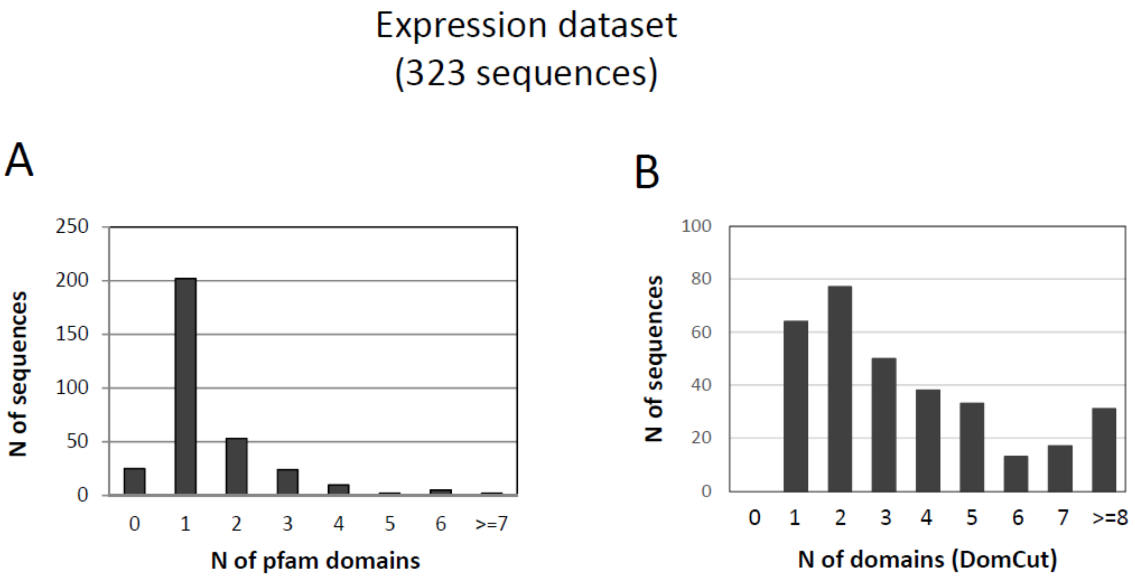

Figure S2

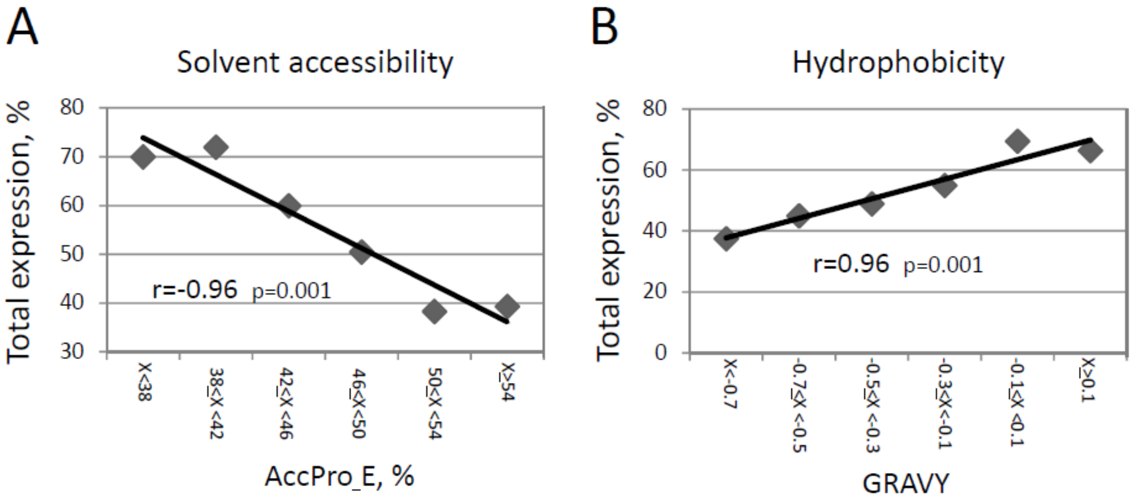

Figure S3

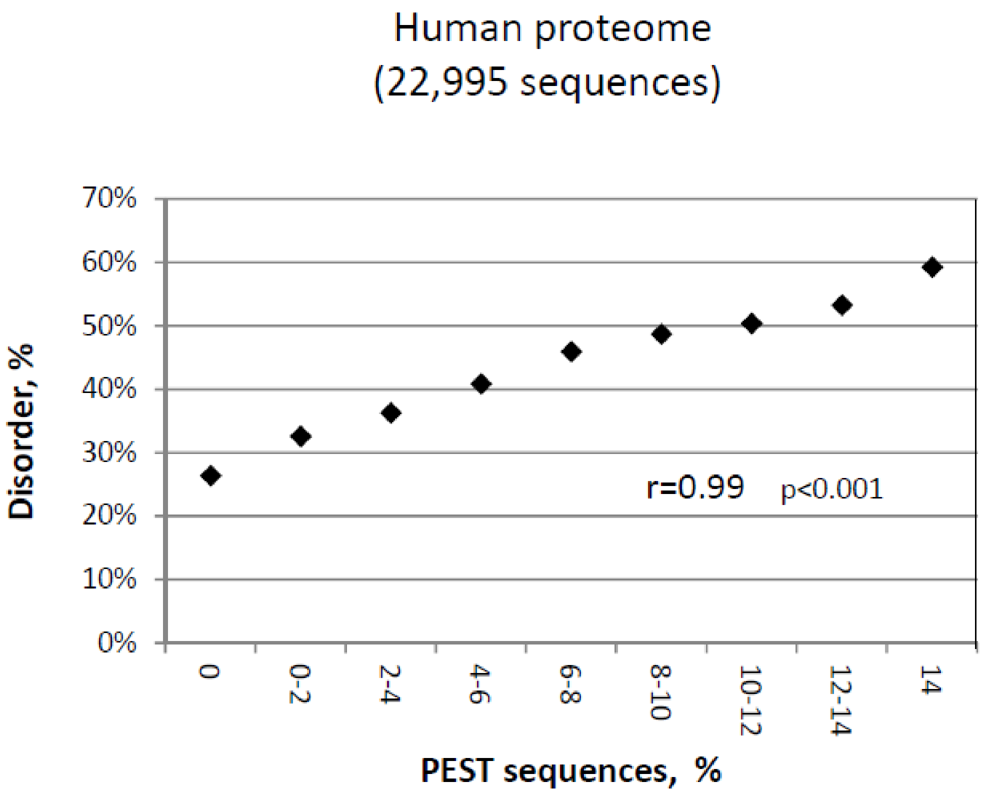

Figure S4

Supplementary Figure Legends

Figure S1. Correlation between disorder degree and number of protein domains. Positive correlation between disorder degree and number of protein domains was observed in the expression dataset (A) and in whole human proteome (B). The number of protein domains was predicted using the DomCut algorithm. Pearson's pairwise correlation coefficients and their statistical significance are indicated in the panels.

Figure S2. Distribution of expression dataset proteins according to the number of predicted structural domains. Number of structural domains in the expressed proteins was predicted with either pfam (A) or DomCut (B) algorithms.

Figure S3. Correlations of detectable protein expression with solvent accessibility and hydrophobicity. Negative correlation between the detectable protein expression and solvent accessibility (A) and positive correlation between the detectable protein expression and hydrophobicity (B) observed in the expression dataset. Pearson's pairwise correlation coefficients and their statistical significance are indicated in the panels.

Figure S4. Correlation between PEST and disorder contents in human proteome. Strong positive correlation between PEST and disorder contents was observed in whole human proteome. Pearson's pairwise correlation coefficient and its statistical significance are indicated.
